# Current Genetic Structure Analysis of Leopard Cats Reveals a Weak Disparity Trend in Subpopulations in Beijing, China

**DOI:** 10.3390/biology11101478

**Published:** 2022-10-09

**Authors:** Yang Teng, Jing Yang, Long-Fei Ju, Wen-Hua Huang, Xin Zhang, Fu-Li Gao, Wei-Dong Bao

**Affiliations:** 1 College of Biological Sciences and Technology, Beijing Forestry University, Beijing 100083, China; 2 Institute of Zoology, Chinese Academy of Sciences, Beijing 100084, China; 3 Ecological Environment Management Center of Pearl Spring Township, Beijing 102107, China; 4 Management Office of Yunmengshan Nature Reserve, Beijing 101513, China

**Keywords:** leopard cat, microsatellite DNA, genetic diversity, genetic structure differentiation

## Abstract

**Simple Summary:**

Habitat fragmentation is an important factor leading to the decline in the leopard cat population in Beijing. Habitat loss may further result in population shrinkage, which increases the risk of inbreeding and genetic decline. To reveal the segregation effects of highway construction and infrastructure expansion on population genetic variation, this study analyzed the genetic structure of leopard cats in five nature reserves in the mountain surroundings of Beijing. The results showed that a mild disparity trend exists in Baihuashan and Songshan subpopulations, due to habitat segregation and dispersal difficulties. We suggest that the genetic structures of the leopard cat population be monitored every 5 years to detect any changes. If needed, individuals can be artificially exchanged among different subpopulations to maintain the viability of this wild cat in Beijing.

**Abstract:**

In the face of habitat shrinkage and segregation, the survival of wild cats looks bleak. Interpreting their population genetic structure during habitat fragmentation is critical in planning effective management strategies. To reveal the segregation effects of road construction and human settlements on the population genetic structure, we analyzed non-invasive fecal DNA samples from leopard cats (*Prionailurus bengalensis*) from five nature reserves in mountainous areas around Beijing. We focused on microsatellite markers. A total of 112 individual leopard cats were identified among 601 samples of scat, and moderate population genetic diversity was detected. Microsatellite-marker-based genetic differentiation (Fst) and gene flow (Nm) showed a weak trend toward discrepancies in the Baihuashan and Songshan subpopulations, which indicated habitat fragmentation effects on individual dispersal. Because the segregated subpopulations may suffer a high risk of genetic diversity loss, we suggest that their genetic structure be monitored with more molecular markers to detect any changes, and that female individuals be artificially introduced as needed to maintain the viability of the leopard cat subpopulations in Beijing.

## 1. Introduction

Variation in population genetic structure can influence the interactions between a species and its environment, and populations with higher genetic diversity are more adaptable to risks brought by changing environments [1]. Moreover, genetic diversity may reflect species’ evolutionary potential, and it can supply important information about their current status and conservation strategies to be implemented [2]. When habitat fragmentation occurs, wild animals are threatened by population isolation and genetic loss that may increase the risk of extinction among segregated populations [3]. Thus, analyses of genetic structure are becoming an important part of effective conservation [4]. Studies on genetic variation may identify subpopulations’ differentiations, which would help create different management units to maintain the long-term survival of local populations [5,6]. 

Although small in number, wild feline species are important predators with various body types, diverse dietary habits, and great adaptability to the surrounding environment. These species play key roles in the natural ecosystem [7,8]. As a result of severe disturbance due to human activity, suitable habitats for wild cats are gradually being lost, and their prey are also decreasing, which further threatens their survival [9]. Most feline species are secretive and highly vigilant, which makes them very difficult targets for field research and conservation [10,11]. With the rapid development of non-invasive sampling among wild animals, fecal samples are becoming informative objects in research on wild cats [12,13]. Sex ratio determination, individual identification, and relatedness analysis enable the accurate evaluation of population genetic diversity and contribute much to the conservation of wild cats [14,15]. 

The leopard cat (*Prionailurus bengalensis*) is a small wild cat native to and widely distributed in East Asia, South Asia, and Southeast Asia [16]. This small cat appears almost everywhere in China, except at high altitudes in the Tibetan plateau and in the severe dry lands of the northwest [17]. Although it is assessed as Least Concern for China in the International Union for Conservation of Nature (IUCN) Red List of Threatened Species [18], the leopard cat population is decreasing as a result of habitat loss and illegal hunting [19]. It is listed in the second category of the CITES Appendices to strengthen protection [20]. Moreover, subspecies of the leopard cat are classified differently in the IUCN Red List; for example, *P. b. rabori* is considered Vulnerable [21] and *P. b. iriomotensis* as Critically Endangered [22]. The leopard cat in China is classified as Vulnerable [23].

Most genetic studies of the leopard cat are from East Asia. The populations living on the islands of Japan have been classified into two subspecies: *P. b. euptilurus* and *P. b. iriomotensis* [24,25]. A study on their population genetic structure revealed low levels of DRB allelic variation in MHC class II genes among subpopulations from the islands of Tsushima and Iriomote in Japan [26], which are in line with findings of decreased genetic diversity, based on the DNA of the mitochondrial control region [27]. 

Studies of leopard cats from Korea using microsatellite markers showed that this species had lower genetic diversity than 12 other feline species in the world [28] and other mammalian species in Korea [29]. However, there has been little research of this kind on species in China. One study found five management units for leopard cat populations, based on genetic diversity and phylogenetic analysis using RAPD and variation in mitochondrial DNA [30]. Another study using the mitochondrial DNA of Cyt B and the control region sequence analyzed genetic diversity among populations from five geographic areas and detected independent trends in evolution between northern and southern branches in China [31]. 

It was estimated that there were 1500 leopard cats in the Beijing region in the first national wildlife survey from 1995 to 2000 (Report on resources survey on the terrestrial wild animals in Beijing, unpublished data, 2012). However, few studies focused on leopard cats, except studies on food components by scat residue identification [32,33], camera trapping monitoring, and activity pattern analysis in nature reserves [34,35,36,37]. The only genetic study on mammals in Beijing was on the Chinese goral (*Naemorhedus griseus*), from the Songshan national nature reserve, which detected moderate population genetic diversity [38]. There is a lack of research on the baseline genetic information for the conservation of mammalian species in Beijing. Thus, we aimed to (1) clarify the genetic background of the leopard cat in Beijing through non-invasive fecal sampling and (2) detect genetic differentiation among the subpopulations due to the segregation effects of infrastructure development and the expansion of human settlements. Given the high dispersal ability of the leopard cat and the fact that the influences of infrastructure development have only been at play for several decades, we assumed that there would be no genetic discrepancy among the subpopulations sampled.

## 2. Materials and Methods

### 2.1. Sampling Procedure and DNA Extraction

**Figure 1:**
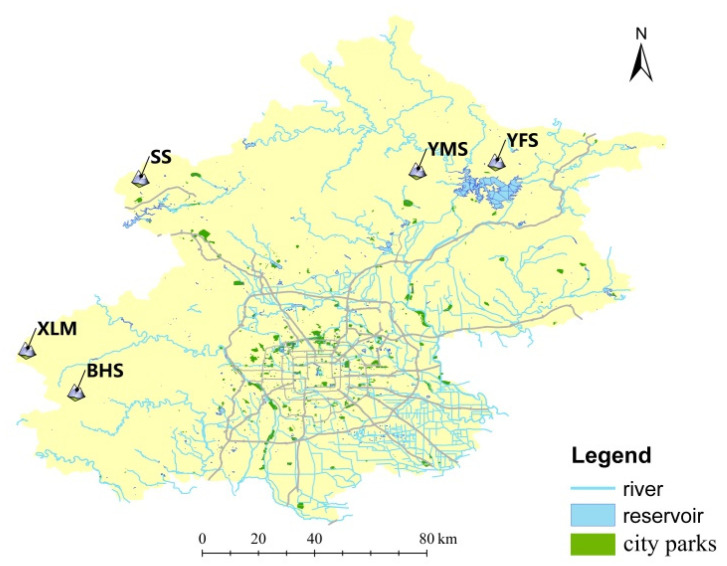
Fecal sampling areas for analyses of the genetic structure of leopard cats in Beijing.

We used the non-invasive analytical method and did not violate animal ethics issues. From September 2017 to October 2018, we collected 601 fecal samples of suspected leopard cat origin by surveying transect lines from the Songshan (*n* = 315), Yunmengshan (*n* = 43), Yunfengshan (*n* = 45), Xiaolongmen (*n* = 75), and Baihuashan (*n* = 123) nature reserves in Beijing (hereafter, SS, YMS, YFS, XLM, and BHS, respectively; Figure 1). Fecal samples were put into sealed plastic bags, fixed with ethanol, and maintained at −20 °C in the laboratory. We extracted the total DNA from the fecal samples using the Stool DNA Kit (D4015-01; Omega, Dorivalle, GA, USA), according to the manufacturer’s protocol. The multiple-tube approach was used to obtain the host DNA by sampling three to five surface parts of one scat, and after the DNA had been extracted separately, they were mixed into one tube to obtain enough DNA for testing the quality using an ultra-micro spectrophotometer (NanoDrop One; Thermo Scientific, Waltham, MA, USA), which was then used in the following analysis. 

BHS represents the Baihuashan reserve, XLM represents the Xiaolongmen reserve, SS represents the Songshan reserve, YMS represents the Yunmengshan reserve, and YFS represents the Yunfengshan reserve. They are the same designations in the following figures.

### 2.2. Species and Sex Identification

The universal primer of the carnivore species ATP6 hypervariable region of mitochondrial DNA was used in species identification [39]. PCR amplification was set to the following conditions: an initial denaturation at 94 °C for 5 min; thirty-five cycles at 94 °C for 30 s, at 60 °C for 30 s, and at 72 °C for 45 s; and final extension at 72 °C for 8 min. Each 30 µL PCR reaction volume contained 15 µL of Premix Ex Taq enzyme (Takara Biomedical Technology, Beijing, China), 0.2 µL of bovine serum albumin (BSA), 1 µL of forward or reverse primer, and 2 µL (~50 ng) of genomic DNA. The PCR products were purified before Sanger sequencing, and afterward, one sequence was obtained (TsingKe Biotech, Beijing, China). The species was identified from a BLAST search of the NCBI database according to the degree of sequence matching. Each experiment included a positive and negative control (DNase/RNase-free deionized water template, rather than DNA).

The zinc-finger regions of the X and Y chromosomes were used to identify the sex of the leopard cat from the fecal samples (Table 1) [40]. The PCR products of the female samples were 165 bp, and the products of the male samples were 162 and 165 bp. The 20 µL PCR reaction volume included 10 µL of Premix Ex Taq enzyme (Takara Biomedical Technology), 0.2 µL of BSA, 0.8 µL of forward or reverse primer, and 2 µL (~50 ng) of genomic DNA. The PCR conditions were an initial denaturation at 95 °C for 10 min; forty cycles at 95 °C for 30 s, at 58 °C for 40 s, and at 72 °C for 30 s; and final extension at 72 °C for 8 min. Genotyping of the samples was performed with an ABI 3730xl DNA analyzer (Applied Biosystems, Foster City, CA, USA) supplied by TsingKe Biotech. Each sample was analyzed using capillary electrophoresis at least three times until the exact genotype was obtained; samples without amplification products were excluded from the analysis.

### 2.3. Microsatellite Loci Selection and Amplification

We repeatedly tried several reaction conditions and annealing temperatures with the fecal DNA samples, based on 20 pairs of microsatellite loci for leopard cats that were used in different studies [28,29]. Only six of them (Pbe03, Pbe05, Pbe13, Pbe28, Pbe32, and Pbe33) worked properly for our fecal samples (Table 1). The conditions for the PCR reaction were retrieved from Ko et al. 2018 [29]. We genotyped the samples using the ABI 3730xl DNA analyzer supplied by TsingKe Biotech (the forward primer was labeled with FAM dye at the 5′ end). Each sample was analyzed at least three times to reduce error. If no effective DNA was detected from the heavy degradation, the scat was discarded to ensure the reliability of the three genotyping repeats. Samples amplified with all six loci were considered to be successful and were used in the following analysis.

### 2.4. Data Analysis

The microsatellite data were organized in Microsoft Excel, and the MS tools plugin in Excel was used to find matching genotypes in the database. The reliability of the typing results was tested with the implementation criteria [41]. Samples were considered to originate from the same individual if the genotypes of all loci were identical or there was a difference in only one locus [42]. 

We tested the Hardy–Weinberg equilibrium and linkage disequilibrium of each target microsatellite locus using Arlequin v3.5 [43] and Genepop v4.3 [44] with the Bonferroni correction. The number of alleles (Na), effective number of alleles (Ne), observed and expected heterozygosity (Ho and He, respectively), polymorphism information content (PIC), probability of identity (PID), and probability of identity between siblings (PID-sibs) were calculated with GenAlEx v6.5 (Peakall R and Smouse P E, Canberra, Australia) [45]. STRUCTURE v2.3.4 (Pritchard Lab., Palo Alto, CA, USA) was used to verify and analyze the genetic differentiation among leopard cat subpopulations with Bayesian clustering. The Evanno ad hoc ΔK statistic was used to determine the most probable clustering number of subgroups [46]. We set the number of clusters (K) from 1 to 5 (10 times for each K) for accurate assignments of accessions. For each run, a burn-in period of 50,000 iterations and a run length of 500,000 MCMC replications were implemented, and the data were processed with the Harvester online software (http://tayloro.biology.ucla.edu/structureHarvester/) (accessed on 12 September 2019).

The non-Bayesian method, DAPC (Discriminant Analysis of Principal Components), was used to compare to the results from STRUCTURE analysis. This method adopted the R software platform to perform a linear discrimination analysis by running the Adegent data-analysis module and the parameter as the main number of factors was set to N ≤ N/3 (n is the total number of individuals) [47]. 

## 3. Results

### 3.1. Species and Individual Identification

There were 601 carnivore fecal samples collected in the five sampling nature reserves. After a BLAST search of the NCBI database, 550 of the samples were identified as those of leopard cats, with a matching rate of ≥ 98%. Of those 550 samples, 508 were discriminated by sex, with 383 from females and 125 from males. The 508 samples successfully identified by sex were amplified using the microsatellite loci; 96 samples that could not be amplified at the six sites were removed. Micro-Checker detected no null alleles at any of the six loci. After microsatellite-genotype-sharing analysis and sex identification, we identified 53 individuals (33 females and 20 males) in SS, 17 individuals (10 females and 7 males) in YMS, 17 individuals (14 females and 3 males) in YFS, 13 individuals (10 females and 3 males) in XLM, and 12 individuals (9 females and 3 males) in BHS.

### 3.2. Microsatellite Analysis of Genetic Diversity

None of the six microsatellite loci used for the analysis of genetic diversity deviated from the Hardy–Weinberg equilibrium (*p* > 0.05), and no linkage disequilibrium was detected. There were 31 alleles detected for the six microsatellite loci and the allele frequency was unevenly distributed; some of them were null in the five subpopulations (Table 2). 

The average Na of the six microsatellite loci was 3.400; the average Ne was 2.288; the average Ho was 0.392; the average He was 0.514; and the average PIC was 0.449. The combined population probability of identity for PID was 1.2 × 10^−3^ in YMS, while it was over 2.5 × 10^−4^ in other areas, and the PID-sibs was in the 10^−2^ level, which indicated that the six microsatellite loci were reliable for individual leopard cat identification in this study (Table 3).

The Fst values of the leopard cat subpopulations in the five sampling areas ranged from 0.011 to 0.082; those Fst values larger than 0.05 were the BHS and SS, BHS and YFS, YMS and SS, and BHS and YMS pairs, showing a mild genetic differentiation among these subpopulations (Table 4). Meanwhile, the Nm was in a large range from 2.799 to 22.478, among which the pairs of BHS and SS, BHS and YFS, YMS and SS, and BHS and YMS were relatively small, displaying normal gene flow for these subpopulations.

**Figure 2:**
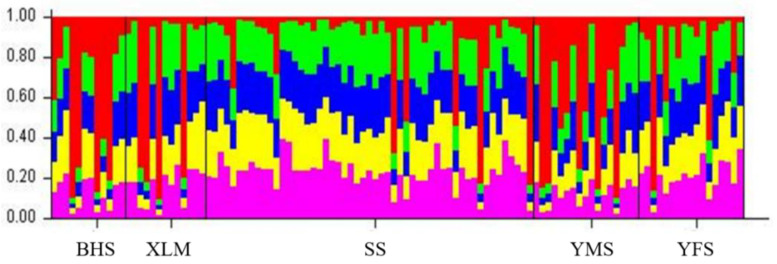
Genetic structure based on microsatellite genotypes in leopard cat subpopulations in the five sampling areas (k = 5). BHS represents Baihuashan in red, XLM represents Xiaolongmen in yellow, SS represents Songshan in green, YMS represents Yunmengshan in magenta, and YFS represents Yunfengshan in blue.

The results of the STRUCTURE analyses showed that when k = 5, the average value of ln-likelihood was higher (when k = 1, ln-likelihood = −1371.9; when k = 2, ln-likelihood = −1291.3; when k = 3, ln-likelihood = −1255.9; when k = 4, ln-likelihood = −1241.7; when k = 5, ln-likelihood = −1233.5). There was no significant genetic differentiation among the subpopulations in the five sampling areas. This indicates that the leopard cats in Beijing share more common genetic background and less genetic differentiation in nuclear DNA diversity (Figure 2).

**Figure 3:**
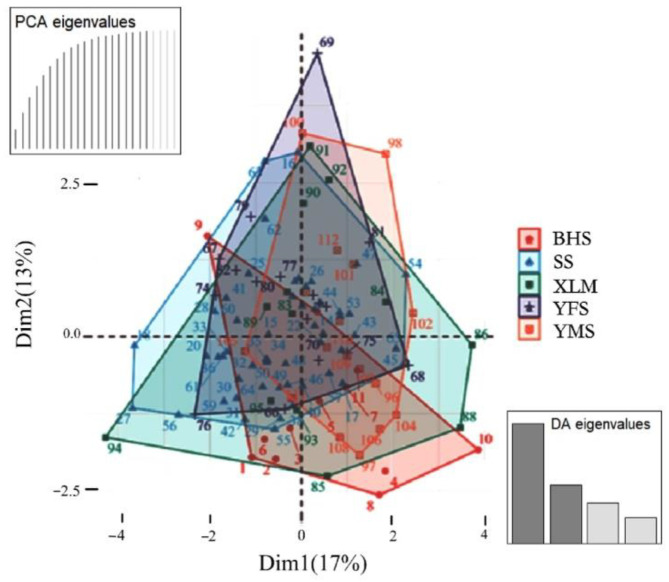
Genetic similarity based on DAPC analysis of microsatellite genotypes in leopard cat subpopulations in the five sampling areas.

The results of the DAPC analysis showed that there was no significant genetic differentiation among the five subpopulations (Figure 3). However, if individual 9 was excluded (dot 9 in the upper left of the plot), most of the individuals in the BHS group were clustered in the lower right, which may indicate that this subpopulation intended to separate from other groups. 

BHS represents Baihuashan in red, SS represents Songshan in blue, XLM represents Xiaolongmen in green, YFS represents Yunfengshan in gray, and YMS represents Yunmengshan in orange.

## 4. Discussion

Accurate evaluation of the population abundance and sex structure is critical to assessing the current status and future development of local populations [48]. From large samples of scat, we identified 112 leopard cats from five nature reserves in mountainous habitats around Beijing. Most individuals were from the SS reserve. The 53 leopard cats in the 45 km^2^ SS reserve [49] may approximate the real state of the population, whereas the abundance in other reserves may be underestimated, due to the lack of successfully DNA-extracted scat samples. 

The sex ratio is an important indicator of the population structure and development trends for the effective management of endangered species [50]. There was a general trend toward a female-biased sex ratio in the five sampling areas (the female-to-male ratio was 1.65:1 at SS, 1.43:1 at YMS, 4.67:1 at YFS, 3.33:1 at XLM, and 3:1 at BHS). This is in line with the female-biased sex structures of other feline species, such as the jungle cat (*Felis chaus*) in India at 1.59:1 [51]. This phenomenon is more obvious among large cats such as the snow leopard (*Panthera uncia*) and the African lion (*Panthera leo*) [52,53] because males die at higher rates than females from injuries during hunting, dispersal, and individual competition [52,54]. However, this female-biased sex ratio may help the population recover quickly from low numbers [55]. Our study provides valuable reference information for the future monitoring of the leopard cat sex ratio in the Beijing region.

### 4.1. Genetic Structure Based on Microsatellite Diversity

Analyzing the genetic structure of local populations through molecular approaches may reveal clues to their genetic diversity and adaptability to the environment [56]. Microsatellites are frequently selected as nuclear markers for their high polymorphism, low requirements for DNA in PCR amplification, and accuracy in assessing population genetic diversity [28]. The combined population probabilities of identity, PID and PID-sibs, were in the levels of 10^−4^ and 10^−2^, respectively. Considering there were less than 100 wild cats in each sampling area [57], the six microsatellite loci were reliable in identifying leopard cat individuals in our study [58]. The average PIC at all six microsatellite loci was above 0.4 (Table 3), which indicated the moderate diversification of the alleles according to the standards of Botstein [59]. In this study, the average effective number of alleles (Ne = 2.288) was lower than the index for leopard cats in Korea (Na = 3.8) [29] but higher than that of the Iriomote leopard cat population in Japan (Na = 1.33) [26]. Moreover, the average observed heterozygosity was lower than for leopard cats in Korea (Ho = He = 0.41) [29], but the average expected heterozygosity was higher (Table 3), and, compared to the Tsushima leopard cat population in Japan (Ho = 0.77, He = 0.66) [26], our parameters were much lower. Thus, total genetic diversity was moderate for the leopard cats in this study, in concordance with their wide distribution and small population size in the Beijing region [57]. 

### 4.2. Population Genetic Differentiation

Habitat loss and fragmentation increase the chance for inbreeding and pose an extinction risk for the local populations, which are becoming major threats to biodiversity conservation [53]. In general, wild animals must have a high rate of dispersal to reduce this risk and maintain a diversified population genetic structure [14]. Based on the analysis of the diversity using microsatellite loci, we found the genetic differentiation index of Fst values was larger than 0.05 for BHS and SS (0.082), BHS and YFS (0.072), YMS and SS (0.057), and BHS and YMS (0.053), indicating a moderate genetic differentiation among these subpopulations. However, the values of Nm indicate a normal gene flow among the subpopulations, although the value pairs of BHS and SS (2.799), BHS and YFS (3.222), YMS and SS (4.136), and BHS and YMS (4.467) were relatively small (Table 4). Meanwhile, the STRUCTURE diagram and DAPC analysis revealed the five sampling groups shared most of the ancestral gene, indicating no obvious genetic discrepancy (Figure 2 and Figure 3). Our results implied that more work is needed to clarify the genetic discrepancy for these separated subpopulations, such as by using mitochondrial markers. However, cautions regarding the effects of female philopatry should be taken into consideration, as the variations in the mitochondrial DNA were mainly dominated by the maternal lineages. For example, female philopatry detected in the Scandinavian brown bear (*Ursus arctos*) demostrated low number of mtDNA haplotypes and high microsatellite diversity among four analyzed subpopulations. It is suggested that this is a result of gene flow mediated by male dispersal and geographical distance [60]. Similar outcomes were detected in an Australian bird (the eastern yellow robin *Eopsaltria australis*) [61]. Thus, if the mitochondrial markers be applied in leopard cat in Beijing, enough samples in different sex should be included to clarify the influence of sex biased dispersal.

## 5. Conclusions

From the point of view of conservation genetics, the Beijing region is in a relatively narrow geographic range with no marked differences in weather or altitude. In addition, the leopard cat has a strong dispersal ability. We postulated at the beginning of our study that there would be no genetic differentiation among subpopulations. However, the mild discrepancy trend in the BHS and SS subpopulations was different from our expectation. We assume that this is due to female-dominated philopatry traits of the leopard cat, as well as segregation effects from natural rivers, major roads, and the expansion of human residential sites. Thus, we suggest that greater attention should be paid to the BHS and SS subpopulations in future monitoring programs, and they should each be taken as an independent conservation unit for the planning of further conservation strategies. If needed, female individuals from other areas could be introduced to maintain the integrity of genetic diversity. We also suggest further field sampling and more molecular markers be utilized to obtain a clearer genetic variation of the leopard cat population in the Beijing region.

## Figures and Tables

**Table 1:** Primer pairs for amplifying the sex chromosomes and microsatellites of the leopard cat.

Locus	Primer Sequence (5′-3′)	Ta (°C)	Size (bp)
Zn-finger	F: AAGTTTACACAACCACCTGG	56	X162/Y165
	R: CACAGAATTTACACTTGTGCA		
Pbe03	F: ^M13F^CTGCCTTTGACTGCTCCAC	58	131–163
	R: TGCTTACCATGTGACCTCC		
Pbe05	F: ^M13F^TCACCTCTGGGCTCTTG	60	181–193
	R: AGGGACACGGAAAGGCATC		
Pbe13	F: ^M13F^TGCGGATGTTGGGAAAGAAC	60	210–218
	R: AGGCCGAGACCAGTTAAGG		
Pbe28	F: ^M13F^GGGAGACCTTGCCTCATTTC	56	233–241
	R: TGCTTCCCTAACAGGCATC		
Pbe32	F: ^M13F^AGCACTAGGCCAGAACACC	64	174–178
	R: CCAGACCCTCTTTGCCTTG		
Pbe33	F:^M13F^AGAGGCACTTGGAGTTAGGG	58	248–252
	R: GAG TCGGCA AACCTGGAAC		

The sequence of M13F is 5′- CACGACGTTGTAAAACGAC -3′ added to the 5′ end of the forward primer labeling with 5-HEX for Pbe03, 05, and 13; 5-FAM for Pbe28, 32, and 33.

**Table 2:** Allele frequencies of six microsatellites in leopard cat subpopulations in the five sampling areas.

Microsatellites Loci	Allele	Allele Frequency				
		SS	YMS	YFS	XLM	BHS
Pbe03	131	0.142	0.059	0.088	0.115	0.042
	135	0.094	0.147	0.000	0.038	0.167
	139	0.057	0.000	0.059	0.000	0.292
	143	0.330	0.559	0.353	0.500	0.417
	147	0.019	0.000	0.059	0.000	0.000
	151	0.113	0.000	0.059	0.000	0.000
	155	0.038	0.000	0.059	0.000	0.000
	159	0.179	0.235	0.324	0.346	0.083
	163	0.028	0.000	0.000	0.000	0.000
	131	0.142	0.000	0.000	0.000	0.000
Pbe05	177	0.000	0.000	0.000	0.154	0.000
	181	0.274	0.500	0.118	0.192	0.250
	185	0.698	0.500	0.882	0.654	0.708
	189	0.000	0.000	0.000	0.000	0.042
	193	0.028	0.000	0.000	0.000	0.000
Pbe13	202	0.000	0.000	0.000	0.115	0.083
	206	0.038	0.000	0.029	0.000	0.000
	210	0.368	0.000	0.176	0.154	0.083
	214	0.377	0.588	0.471	0.500	0.625
	218	0.208	0.412	0.324	0.077	0.125
	222	0.009	0.000	0.000	0.154	0.083
Pbe28	233	0.028	0.235	0.088	0.231	0.542
	237	0.623	0.441	0.500	0.538	0.208
	241	0.349	0.324	0.412	0.231	0.250
Pbe32	170	0.019	0.029	0.000	0.000	0.000
	174	0.292	0.176	0.324	0.269	0.250
	178	0.679	0.794	0.676	0.731	0.750
	182	0.009	0.000	0.000	0.000	0.000
Pbe33	240	0.000	0.000	0.059	0.000	0.000
	248	0.217	0.294	0.294	0.269	0.125
	252	0.783	0.706	0.647	0.731	0.875

SS represents Songshan reserve, YMS represents Yunmengshan reserve, YFS represents Yunfengshan reserve, XLM represents Xiaolongmen reserve, and BHS represents Baihuashan reserve. They are the same designations in the following tables.

**Table 3:** Genetic diversity and probability of identity of six microsatellite loci detected in leopard cat subpopulations in the five sampling areas.

Microsatellite Loci	Microsatellite Index	SS (N = 53)	YMS (N = 17)	YFS (N = 17)	XLM (N = 13)	BHS (N = 12)
Pbe03	Na	9.000	4.000	7.000	4.000	5.000
	Ne	5.300	2.546	3.986	2.600	3.388
	Ho	0.698	0.588	0.588	0.462	0.583
	He	0.811	0.607	0.749	0.615	0.705
	PIC	0.819	0.554	0.713	0.544	0.655
	PID	0.0576	0.2074	0.0993	0.2188	0.1360
	PID-sibs	0.3587	0.4982	0.4003	0.4970	0.4316
Pbe05	Na	3.000	2.000	2.000	3.000	3.000
	Ne	1.776	2.000	1.262	2.048	1.767
	Ho	0.415	0.294	0.235	0.385	0.583
	He	0.437	0.500	0.208	0.512	0.434
	PIC	0.441	0.375	0.186	0.458	0.369
	PID	0.03908	0.3750	0.6794	0.2919	0.3850
	PID-sibs	0.6292	0.5938	0.8086	0.5671	0.6292
Pbe13	Na	5.000	2.000	4.000	5.000	5.000
	Ne	3.102	1.940	2.792	3.159	2.341
	Ho	0.642	0.353	0.529	0.462	0.250
	He	0.678	0.484	0.642	0.683	0.573
	PIC	0.684	0.367	0.575	0.647	0.544
	PID	0.1674	0.3831	0.1955	0.1366	0.2118
	PID-sibs	0.4530	0.6036	0.4780	0.4424	0.5165
Pbe28	Na	3.000	3.000	3.000	3.000	3.000
	Ne	1.960	2.820	2.340	2.522	2.504
	Ho	0.566	0.471	0.412	0.154	0.667
	He	0.490	0.645	0.573	0.604	0.601
	PIC	0.494	0.571	0.481	0.536	0.533
	PID	0.3557	0.1997	0.2739	0.2246	0.2270
	PID-sibs	0.5941	0.4773	0.5321	0.5044	0.5064
Pbe32	Na	4.000	3.000	2.000	2.000	2.000
	Ne	1.827	1.509	1.778	1.649	1.600
	Ho	0.547	0.176	0.294	0.231	0.167
	He	0.453	0.337	0.438	0.393	0.375
	PIC	0.457	0.297	0.342	0.316	0.305
	PID	0.3790	0.4795	0.4120	0.4453	0.4609
	PID-sibs	0.6184	0.7012	0.6341	0.6646	0.6777
Pbe33	Na	2.000	2.000	3.000	2.000	2.000
	Ne	1.515	1.710	1.966	1.649	1.280
	Ho	0.321	0.235	0.294	0.077	0.083
	He	0.340	0.415	0.491	0.393	0.219
	PIC	0.343	0.329	0.415	0.316	0.195
	PID	0.4936	0.4282	0.3347	0.4453	0.6343
	PID-sibs	0.7035	0.6494	0.5880	0.6646	0.7992
Average	Na	4.333	2.667	3.500	3.167	3.333
	Ne	2.580	2.087	2.354	2.271	2.147
	Ho	0.531	0.353	0.392	0.295	0.389
	He	0.535	0.498	0.517	0.534	0.484
	PIC	0.470	0.418	0.452	0.470	0.434
	combined PID	2.5 × 10^−4^	1.2 × 10^−3^	4.7 × 10^−4^	3.9 × 10^−4^	7.3 × 10^−4^
	combined PID-sibs	2.6 × 10^−2^	3.8 × 10^−2^	3.0 × 10^−2^	2.7 × 10^−2^	3.8 × 10^−2^

**Table 4:** Fst and Nm based on analyses of the diversity of microsatellite loci in leopard cat subpopulations in the five sampling areas.

Sampling Area	SS	YMS	YFS	XLM	BHS
SS		4.136	18.981	11.655	2.799
YMS	0.057		4.852	9.366	4.467
YFS	0.013	0.049		22.478	3.222
XLM	0.021	0.026	0.011		6.695
BHS	0.082	0.053	0.072	0.036	

Fst appears below the diagonal, and Nm appears above the diagonal.

## Data Availability

This study did not report any data.
